# Supplementary material for: ICMR’s multistate implementation research study on integration of screening and management of mental and substance use disorders with other non-communicable diseases (ICMR-MINDS) – An implementation research study protocol
Source: PLoS One. 2025 Oct 3;20(10):e0332359. doi: 10.1371/journal.pone.0332359 (PMC12494248; doi:10.1371/journal.pone.0332359)
Supplement: S1 File — (PDF) [file pone.0332359.s002.pdf]

Supplement 1. Abbreviations	
ANM	Auxiliary Nurse Midwife
AAM	Ayushman Arogya Mandirs
AAM-PHC	Ayushman Arogya Mandirs—Primary Health Centers
AAM-SC	Ayushman Arogya Mandirs - Sub Centers
ASHA	Accredited Social Health Activist
CFIR	Consolidated Framework For Implementation Research
CHC	Community Health Center
CHO	Community Health Officer
CKD	Chronic Kidney Disease
COPD	Chronic Obstructive Pulmonary Disease
CVD	Cardiovascular Disease
DM	Diabetes Mellitus
DMHP	District Mental Health Program
FGD	Focused Group Discussion
GAD	Generalized Anxiety Disorder
HR	Human Resource
HTN	Hypertension
ICMR	Indian Council Of Medical Research
IDI	In-Depth Interview
IEC	Information, Education, and Communication
MH	Mental Health
MhGAP	Mental Health Gap Action Program
MPW-M	Multipurpose Health Worker Male
MSUD	Mental and Substance Use Disorders
NCD	Non-Communicable Diseases
PHC	Primary Health Center
PHQ-2	Patient Health Questionnaire 2
PHQ-9	Patient Health Questionnaire 9
RE-AIM	Reach Effectiveness—Adaptation Implementation Maintenance
STL	Screening, Treatment, and Linkage
WHO	World Health Organization
