# Supplementary material for: ICMR’s multistate implementation research study on integration of screening and management of mental and substance use disorders with other non-communicable diseases (ICMR-MINDS) – An implementation research study protocol
Source: PLoS One. 2025 Oct 3;20(10):e0332359. doi: 10.1371/journal.pone.0332359 (PMC12494248; doi:10.1371/journal.pone.0332359)
Supplement: S1 Table — (PDF) [file pone.0332359.s003.pdf]

Assumption	Calculation	Adjusted sample (rounded up)
Base (no DEFF, no non-response)	81	**81**
10% non-response only	81 × 1.10	**90**
DEFF = 1.2 (clustering) only	81 × 1.2 = 97.2	**98**
DEFF = 1.2 + 10% non-response	98 × 1.10 = 107.8	**108**
DEFF = 1.5 (conservative) only	81 × 1.5 = 121.5	**122**
DEFF = 1.5 + 10% non-response	122 × 1.10 = 134.2	**135**
**Note:** Base calculation: n=81 (rounded from 80.7)		
