# Supplementary material for: ICMR’s multistate implementation research study on integration of screening and management of mental and substance use disorders with other non-communicable diseases (ICMR-MINDS) – An implementation research study protocol
Source: PLoS One. 2025 Oct 3;20(10):e0332359. doi: 10.1371/journal.pone.0332359 (PMC12494248; doi:10.1371/journal.pone.0332359)
Supplement: S1 Fig — (PDF) [file pone.0332359.s001.pdf]

Applicable to all adults and adolescents seen by CHO

Referred by ASHA or when asked about their problem, if any one of the symptoms in the Box-1 mentioned by the person/family member/caregiver/friend, then go through this workflow

Box-1 (Common Mental Health Complaints)

1. Feeling sad or having mood swings
2. Feeling nervous, restless or anxious
3. Worried, stressed or fearful
4. Loss of interest in school work,household chores,decline in academic/work, or neglecting usual responsibilities
5. Problems related to sleep/appetite (associated weight gain or loss)/sex
6. Started being withdrawn from friends/activity, not connected to reality
7. Problems in marital/ family/work-related relationships
8. Hearing or seeing things that do not exist
9. False beliefs not shared by others
10. Any action or mention of self harm
11. Aggressive/unusual/agitated behaviour
12. Repetitive washing/cleaning
13. Using substances like alcohol/cannabis/opioid/tobacco or having any other addiction

If patient has come to follow up for any mental health issues proceed to follow up care (management cards)

If any one symptom is present, proceed to assess for a mental health emergency (refer to Box-2).

Box-2
Assess for Mental Health Emergency

Look for emergency signs

1. Appearing affected by alcohol or other substance intoxication (smell of alcohol)
2. Unconscious or extremely lethargic
3. Sign of self harm, self inflicted wound,poisoning
4. Seizure

Ask following questions (in last 2 weeks)

5. False, unshakable/ fixed belief which is not held by others in the family/ community
6. Hearing voices or seeing things that do not exist
7. Recent history of highly agitated or aggressive behaviour
8. Thought of self harm or history of self harm e.g. poisoning,self inflicted wound
9. History of seizure in past 24 hours
10. Unprovoked violence

If any one symptom is present, proceed to emergency management.

Possible Mental Health Emergency

- Provide psychological first aid
- Immediate referral to mental health emergency care at district level/medical college
- Ensure the person is accompanied with family/friend/caregiver to referral centre

If no to all, proceed through all the boxes below (Box 3, Box 4, and Box 5).

Box-3
Assess for Depression

1. Apply Patient Health Questionnaire (PHQ-9)
2. If score ≥1 for Q-9, on suicidal thought - **go to Possible Mental Health Emergency Management**

Assess	Classify	Identify Management
• Day to day functioning affected or PHQ-9 ≥ 15	Possible Severe Depression	• Psychoeducation<br>• Refer to Mental Health trained Medical Officer/Psychiatrist
• PHQ-9 score 10-14	Possible Moderate Depression	• Teleconsultation - 14416 (if not possible, refer to mental health trained MO<br>• Counselling by CHO using module (psychoeducation, self help strategies-relaxation exercise, lifestyle modification,yoga, meditation, avoid substance use)
• PHQ-9 score 5-9	Possible Mild Depression	• Counselling by CHO using module (psychoeducation, self help strategies-relaxation exercise, lifestyle modification, yoga, meditation, avoid substance use)<br>• Repeat screening after 2 weeks(if score increases or no improvement in symptoms, follow management as per above mentioned box)
• PHQ-9 score 0-4	Depression Unlikely	• Wellness promotion activities (self help strategies- relaxation exercise, lifestyle modification,yoga, meditation, avoid substance use)

Box-4
Assess for Anxiety

1. Apply Generalized Anxiety Disorder Questionnaire (GAD-7)

Assess	Classify	Identify Management
• Day to day functioning affected or GAD-7 score ≥ 15	Possible Severe Anxiety	• Refer to Mental Health trained Medical Officer/Psychiatrist<br>• Psychoeducation
• GAD-7 score 10-14	Possible Moderate Anxiety	• Teleconsultation-14416 (if not possible then refer to a mental health trained MO)<br>• Counselling by CHO using module (psychoeducation, self help strategies- progressive muscle relaxation, breathing exercise, lifestyle modification, yoga, meditation, avoid substance use)
• GAD-7 score 5-9	Possible Mild Anxiety	• Counselling by CHO using module (psychoeducation, self help strategies-progressive muscle relaxation, breathing exercise, lifestyle modification, yoga, meditation, avoid substance use)<br>• Repeat screening after 2 weeks (if score increases or no improvement in symptoms, follow management as per above mentioned box)
• GAD-7 score 0-4	Anxiety less unlikely	• Wellness promotion activities (self help strategies, breathing exercise, yoga, meditation, avoid

Box-5
Assess for Substance Use

1. Do you consume tobacco?
2. Do you currently consume alcohol or any other addictive drug or substance? If yes, please proceed to the next question. If no, please follow the green row on the right.
   2.1 How often do you consume the above substance? (Daily/ weekly or monthly)
   2.2 Have you experienced any physical/ health problems to the consumption of the above substance? Or any financial/ family/ legal problems?
   2.3 Have you not been able to go to your job/profession or fulfil household duties due to the consumption of the above substance?

Assess	Classify	Identify Management
• If yes to question -1	Tobacco use	• Psychoeducation<br>• Refer to TCC Counsellors (CHC, District) / Tobacco quit line (1800112356)<br>• Follow-up after 15 days
• Daily/ weekly consumption and yes response to question 2.2 or 2.3	Problematic use of alcohol/ opioids/ cannabis/ other substances	• Psychoeducation<br>• Refer to Psychiatrist/ Mental Health trained Medical Officer for problematic use of alcohol/ addictive drug/ other psychoactive substance<br>• Follow-up after 15 days
• Consumes substance once in a month or less and no response to question 2.2 or 2.3	Low risk of current problematic use	• Brief intervention by CHO<br>• Counselling by CHO using module (psychoeducation, self help strategies-breathing exercise, lifestyle modification, yoga, meditation)<br>• Follow-up after one month
• No history of substance use		• Wellness promotion activities (self-help strategies- breathing exercise, lifestyle modification, yoga, meditation)

If there is more than one probable diagnosis, management will be conducted according to the highest category (red, orange, yellow, green)
